# How important is the choice of the nutrient profile model used to regulate broadcast advertising of foods to children? A comparison using a targeted data set

**DOI:** 10.1038/ejcn.2013.112

**Published:** 2013-06-26

**Authors:** P Scarborough, C Payne, C G Agu, A Kaur, A Mizdrak, M Rayner, J C G Halford, E Boyland

**Affiliations:** 1 British Heart Foundation Health Promotion Research Group, Department of Public Health, University of Oxford, Oxford, UK; 2 Department of Experimental Psychology, University of Liverpool, Liverpool, UK

**Keywords:** nutrient profile, advertising, nutrition, regulation

## Abstract

**Background/Objective:**

The World Health Assembly recommends that children's exposure to marketing of unhealthy foods should be reduced. Nutrient profile models have been developed that define ‘unhealthy' to support regulation of broadcast advertising of foods to children. The level of agreement between these models is not clear. The objective of this study was to measure the agreement between eight nutrient profile models that have been proposed for the regulation of marketing to children over (a) how many and (b) what kind of foods should be permitted to be advertised during television viewed by children.

**Subjects/Methods:**

A representative data set of commercials for foods broadcast during television viewed by children in the UK was collected in 2008. The data set consisted of 11 763 commercials for 336 different products or brands. This data set was supplemented with nutrition data from company web sites, food packaging and a food composition table, and the nutrient profile models were applied.

**Results:**

The percentage of commercials that would be permitted by the different nutrient profile models ranged from 2.1% (0.4%, 3.7%) to 47.4% (42.1%, 52.6%). Half of the pairwise comparisons between models yielded kappa statistics less than 0.2, indicating that there was little agreement between models.

**Conclusions:**

Policy makers considering the regulation of broadcast advertising to children should carefully consider the choice of nutrient profile model to support the regulation, as this choice will have considerable influence on the outcome of the regulation.

## Introduction

Over the past three decades, childhood obesity has increased in prevalence in almost every country for which data are available.^1^ Obesity in childhood is significantly associated with risks to physical and mental health,^2^ and its increasing prevalence is therefore a matter of grave public health concern. Obese children are more likely to become obese adults.^3^ The global obesity epidemic currently affects over 10% of the adult population worldwide.^4^ Television advertising is known to be an important influence on children's preferences for food and drinks^5, 6, 7^ (henceforth we include drinks in the term ‘foods'). Television advertising continues to be the most dominant promotional channel, and the food products promoted have an undesirable nutritional profile.^8^ Such evidence has supported calls for the regulation of broadcast advertising of foods to children, and in 2010 the World Health Assembly passed a resolution endorsing WHO recommendations to ‘reduce both the exposure of children to, and power of, marketing of foods high in saturated fats, trans-fatty acids, free sugars or salt', and urging member states to implement the recommendations.^9^

Nutrient profile models classify or rank foods according to their nutritional composition for reasons related to prevention of disease and promotion of health.^10^ Nutrient profile models can be used to support the regulation of food advertising to children by identifying those foods that should (or should not) be advertised to children. Many nutrient profile models have been developed by academics, health charities, national governments and the food industry, some of which have been designed to regulate the broadcast advertising of foods. It is not clear how much influence the choice of a nutrient profile model could have on the outcomes of regulation. This paper addresses the following research question: do nutrient profile models designed for the regulation of broadcast advertising of foods to children agree on (a) the proportion of foods that should be advertised and (b) the type of foods that should be advertised? In this paper we compare nutrient profile models designed for the regulation of broadcast advertising of foods to children using a representative data set of food commercials that were broadcast in the UK in 2008, before the complete introduction of regulations on the broadcast advertising of foods to children in 2009.^11^

## Materials and methods

### Development of a representative data set of food commercials during television viewed by children

The initial data set of food commercials was developed from a sample broadcast in 2008 on the 14 commercial television channels most popular among children aged 4–16 years in the UK.^12^ The channels included those predominately broadcasting general family (for example, ITV, Sky One), sports (for example, Sky Sports One), dedicated children's (for example, Nickelodeon, Boomerang) and music/related programmes (for example, MTV, Smash Hits). The sample was taken from television recordings on one week day and one weekend day every month (from 0600 until 2200 hours) during the study period (January–December 2008). The data set consisted of the name of the food, brand or company advertised and the number of commercials recorded in the sample, and included 455 different commercials broadcast on a total of 18 888 occasions.

Commercials were excluded from the data set if they were (1) for an alcoholic drink or for tea/coffee or chewing gum; (2) for a retailer that provides a broad range of products (for example, a supermarket); (3) for a food product for babies or toddlers; or (4) for weight-loss or weight-gain shakes. A total of 11, 763 commercials remained after these exclusions.

The remaining commercials were categorised as follows: (1) single food items; (2) single brands that include a range of products (for example, Golden Wonder Pot Noodles, which are available in 12 flavours in the UK); and (3) meals that incorporate more than one food item (for example, McDonald's Big Mac Meal). Nutrition data were sourced for these three categories as follows: for single food items, company websites were reviewed and all available nutrition data and recommended serving sizes were taken. When information that is usually available from nutrition information panels was not available from the company web site, food packaging was sourced and nutrition data were extracted. These data were supplemented with nutrition data for a similar food from a UK food composition table of generic foods^13^ (for example, nutrition data for Kerry Cheestrings was supplemented with data on the generic food ‘Cheese, processed slices or block'). For brands, a single product from the range was selected randomly to represent the brand, and nutrition data were sourced as for single food items. For meals, nutrition data for each component of the meal were sourced as for single food items. Using serving size data, the nutrition data were then combined to create weighted-average nutrition data for the meal (including drinks). Throughout, when serving size data were not available from company web sites they were supplemented by a UK serving size guide.^14^ An internal validity exercise was conducted to assess the similarity between Supplementary Data from the generic food composition table and data extracted directly from web sites/ packaging, when both sets of data were available. Correlation (assessed using the Pearson correlation coefficient) between the two sets of data ranged from *r*=0.62 for sodium per 100 g to *r*=0.92 for energy per 100 g, indicating that agreement between the two data sources was strong. For 324 of the 336 included foods, we were able to extract nutritional data from web sites or food packaging.

### Nutrient profile models

Eight nutrient profile models were included in this analysis (see Box 1 for details). The models were developed by national government agencies, academic research groups, consumer groups and commercial organizations, and are either currently used or proposed for use to regulate the advertising of foods to children, either on a voluntary or a mandatory basis. The algorithms for the models were converted to STATA (version 11, StataCorp 2009; College Station, TX, USA) syntax files and checked by three researchers for veracity. The syntax files are included as Supplementary Material.

### Statistical analyses performed

The proportion of commercials (weighted by the number of commercials broadcast) that would be permitted by the different nutrient profile models was calculated, with accompanying 95% confidence intervals assuming a binomial distribution. Pairwise agreement between the models was assessed using Cohen's Kappa statistic, with agreement assessed as follows: 0.21–0.40 ‘fair' 0.41–0.60 ‘moderate' and 0.61–0.80 ‘good'.^25^ All commercials were split into seven categories based on the UK food guide:^26^ bread, cereals and potatoes; fruit and vegetables; meat, fish and alternatives; milk and dairy; fatty and sugary foods; composite foods (foods composed of items from more than one nutritional group, such as pizza); and miscellaneous (foods that did not fit into any of the above categories). The ‘fatty and sugary foods' category was further subdivided into snacks, not snacks and drinks. A full list of the foods and their UK food guide categorizations are available in Supplementary Materials. For each food, the number of models that would permit the food to be advertised on television was calculated.

## Results

Table 1 describes the data set used for this study. Fruit and vegetables were shown in only 2.0% (*n*=257) of commercials, whereas 27.3% (*n*=3217) of commercials featured fatty and sugary foods, mainly snacks and drinks.

Table 2 describes the eight nutrient profile models included in the analysis. No single nutrient or food component is used by all eight models, although fat and sugar are found in some form in each. Sodium is the most common nutrient and is used by all but the Danish model. Note that the criteria for the Danish model state that in some instances an additional criterion for sodium should be used, but it is not clear when the sodium criterion should be applied. For the analyses in this paper we have assumed that the sodium criterion does not apply. Food categories are used by all models, and the number ranges from only two categories (for example, different criteria for food and drink in the UK model) to 20 categories for the EU Pledge Nutrition Criteria. Different threshold levels are applied to foods in different categories; for example, in the EU Pledge model, the sodium criterion is ⩽300 mg/100 g for milk and milk substitutes but ⩽450 mg/100 g for breakfast cereals.

Table 3 shows that the eight models varied greatly in the percentage of foods permitted to be advertised. The Disney model was the strictest, approving just 2.1% (0.4–3.7%) of commercials, followed by the US (Interagency) model, approving 13.5% (9.8%, 17.2%) of the commercials. The most lenient model was the UK model, which permitted 47.4% (42.1%, 52.6%) of the commercials to be broadcast.

Table 4 shows Kappa values indicating the levels of agreement between the models. Individual Kappa values vary greatly with different comparisons. The highest pairwise agreement found was between the Brazilian and UK models (K=0.73), but 14 of the 28 comparisons achieved a Kappa score of <0.20 (the lower threshold for ‘fair' agreement^25^).

**Figure 1:**
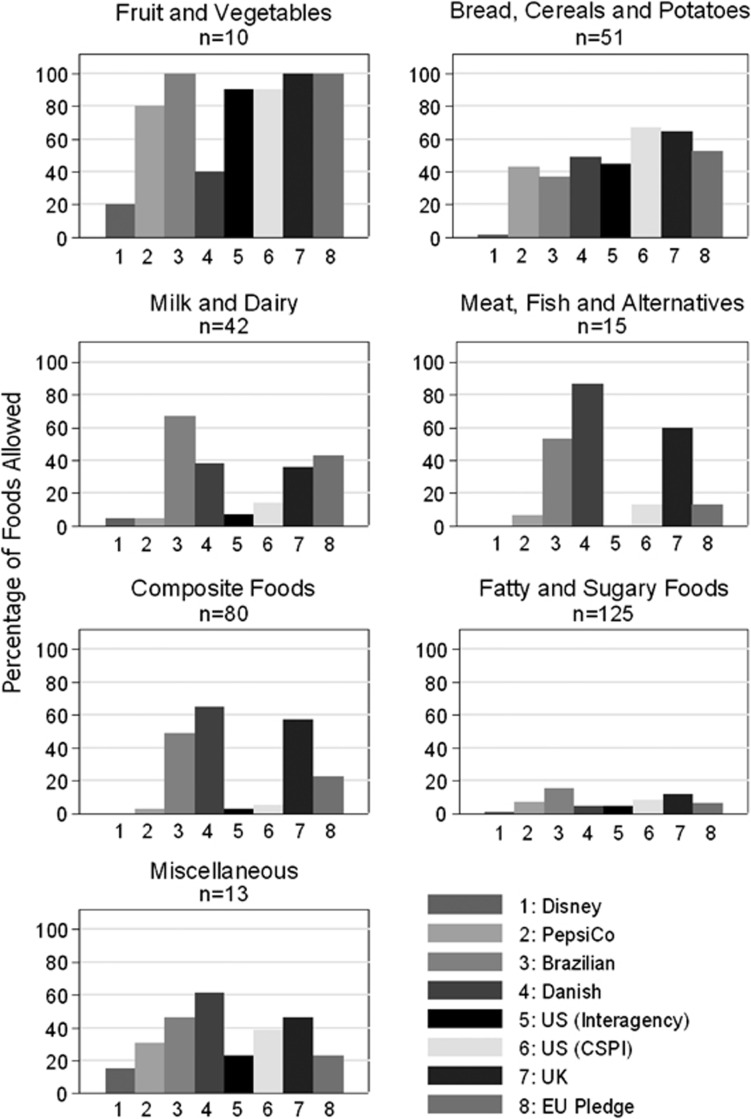
Percentage of commercials allowed to be broadcast by each nutrient profile model by food category.

Figure 1 shows the percentage of food commercials permitted to be advertised by food category, and shows that for some food categories the level of strictness differs considerably between models. For example, in the fruit and vegetables category, the Danish model and the Disney model would allow half and 20% of commercials in the data set to be advertised, respectively, in comparison with other models that would allow a much higher percentage (the UK, Brazilian and EU Pledge models allow all fruit and vegetable products to be advertised). The Danish model was much more lenient in the meat, fish and alternatives category than any other model, allowing more than 80% of the commercials in the data set to be broadcast, compared with <20% for the PepsiCo, Brazilian and CSPI models and none for the Disney and Interagency models. The Disney and Interagency models would also allow fewer commercials for milk and dairy products compared with other models.

In addition, each food was given a score that reflected the number of nutrient profile models that would allow that food to be advertised. Only five of the commercials in the data set would be allowed to be broadcast by all eight models (for Birdseye Garden Peas, Quaker Porridge Oats, Florette Lettuce, Isklar Bottled Water and Drench Bottled Water). In contrast, 38% of the commercials would not be permitted by any of the eight models. This suggests that there is good agreement between the models about which foods should not be permitted to be advertised, but little agreement over which foods should be permitted.

## Discussion

Nutrient profile models that have been developed for the purpose of regulating broadcast advertising of foods to children vary considerably in both the total number of food commercials that they permit (overall strictness) and the type of foods that they permit to be broadcast. Therefore, policy makers considering the regulation of food advertising to children should be careful in their choice of nutrient profile model underpinning the regulation, as this choice will have considerable impact on what commercials children are exposed to.

The eight nutrient profile models compared in this paper, each of which were developed with the purpose of identifying foods that should not be advertised during children's television, showed reasonable agreement about which commercials should not be permitted (for example, fatty and sugary foods—mostly snacks and confectionery). However, there was very little agreement over which commercials should be permitted. The proportion of commercials for breads and cereal products that would be permitted by the models ranged from 2% to 67% for meat based products this ranged from 0% to 87%. Bread and cereal products tend to be rejected on the basis of high sugar levels, whereas meat-based products fail because of not being able to meet fat and/or sodium thresholds. An advantage of using a nutrient profile model to underpin regulation is that it can be applied universally, as opposed to restricting regulation to arbitrarily selected food categories, which allows no scope for product reformulation. This research shows that there is very little agreement between nutrient profile models outside the ‘fatty and sugary foods' category. Hence, although each nutrient profile model provides a categorization for all foods, value judgements about which nutrient profile model will support the regulation will determine the balance of food advertising.

There are at least two questions to consider in this value judgement: (i) how strict should the regulation be? (for example, should it restrict advertising solely to ‘healthy' foods? or should it allow advertising for all foods except ‘unhealthy' foods?) and (ii) which food categories are broadly ‘healthy' or ‘unhealthy'? Almost all of the validation work that has been conducted on nutrient profile models has relied to some degree on similar judgements. Most validation studies have compared food classifications from a nutrient profile model with ‘a standard derived from subjective decisions from nutritionists and dietitians',^19, 21^ or have examined the proportions of foods classified as healthy/unhealthy by a model that make up healthy/unhealthy diets assessed with varying degrees of subjectivity.^20, 27, 28^ To our knowledge, only one study has validated a nutrient profile model against an objective gold standard of prospective health outcomes by categorizing cohort study participants into diet-quality quintile groups based on the nutrient profile model's scores of foods consumed.^29^

The different classifications produced by the models tested in this paper are a result of variations in the way that they have been developed, including the nutrients selected for the model and the number of food categories that the nutrient profile model uses. Ideally, the nutrients selected for use in a nutrient profile model should be directly linked to health outcomes for the target population. Most nutrient profile models categorise foods on the basis of ‘negative' nutrients or food components only, whereas others balance the ‘negative' with ‘positive' nutrients and food components. It has been argued that a small number of food categories are needed for a nutrient profile model on nutritional grounds,^30^ but including too many could lead to manufacturers manipulating food categories for preferable treatment. Conversely, including many food categories provides incentives to the food industry for reformulation.

The analysis reported in this paper is one of the few instances of nutrient profile models being compared using a data set of foods that is representative of a population that is appropriate and relevant to the design of the nutrient profile models. Previous studies that have compared the classifications of nutrient profile models^21, 19, 31^ provide useful information about how different nutrient profile models classify foods, but the results have not been representative of any specific policy scenario.

The data set used here is based on UK commercials, and the nutritional composition data are from UK foods; hence, the results may not be directly applicable to other countries where either the content of food advertising or the nutritional composition of branded foods may be different. However, other research from outside the UK has also found that the majority of foods and food products promoted to children are energy dense, high fat, sugar, and/or salt, in sharp contrast to national and international recommendations.^8^ The data set was based on children's actual viewing habits, rather than including only programmes designed specifically for children (that is, it included programmes such as football matches and general entertainment programmes, which are watched by a large number of children but are not covered by the current UK regulations on television advertising).^11^ Therefore, the results obtained overestimate the actual restriction that advertising regulations using the nutrient profile models would lead to, as regulation tends to cover a narrower programme range or only specific times of day. The importance of the non-nutritional elements of the regulation of food marketing is beyond the scope of this paper, but has been noted elsewhere.^32, 33^

In order to apply the nutrient profile models to the data set of commercials, we needed to supplement it with nutrition data from a generic food composition table, which may not be entirely accurate for the specific branded products included in the data set. It was only possible to check whether the information given in the Supplementary Data was similar to that reported for the specific foods for nutrients for which both sets of data were available, and these checks showed that there was good agreement between the two data sources. Assessment of regression coefficients suggested that the Supplementary Data tended to slightly overestimate the amount of energy, protein, fibre, sugar, saturated fat and sodium, which suggests that the results may overestimate the percentage of ‘unhealthy' foods within the data set; however, the size of this effect was small. The categorization of foods for each nutrient profile model was based on a single researcher's decision based solely on the name of the food, which in the majority of cases was uncontroversial, but some foods may have been miscategorised.

The consistent treatment of commercials for multiple food items in this paper enables comparisons to be made between the different nutrient profile models regardless of the technicalities in the application of the nutrient profile model to more complex advertisements, such as those for multiple products (either comprising a meal, or different products within a range). However, the methods used to assess advertisements for multiple products are not necessarily the methods that regulatory bodies (governmental or otherwise) would use to assess such commercials. For example, regulatory bodies may decide that each product in the advertisement, or element of a meal, must meet the nutrient profile model's criteria. Cases in which food and drink items are advertised together are particularly important, as averaging food and drink items will affect the nutrient density, and nutrient profile models should probably be adjusted accordingly if this technique is to be applied. It is acknowledged that averaging the nutrient content of meals is a limitation of the approach used in this study and may have affected the results by allowing more advertisements for meals than actual regulations.

## Conclusion

There is a public health imperative to enforce regulations to reduce children's exposure to commercials promoting unhealthy foods. The impact of such regulations will depend upon the choice of the nutrient profile model used to support them. It is crucial that policy makers have a clear understanding of how different nutrient profile models designed for similar purposes can classify foods differently. Further research comparing the classifications of nutrient profile models with objective external measures (for example, prospective health outcomes) is warranted.

## Figures and Tables

**Table 1:** The data set of 336 foods advertised, and the frequency of commercials

*Food Category*	*Number of Foods*	*Number of Commercials*	*% of Commercials*
Bread, Cereals and Potatoes	51	2821	24.0
Composite Foods	80	2346	19.9
Fatty and Sugary Foods	125	3217	27.3
Snacks	91	3091	17.8
Not snacks	14	491	4.2
Drinks	20	635	5.4
Fruit and Vegetables	10	234	2.0
Meat, Fish and Alternatives	15	257	2.2
Milk and Dairy	41	2381	20.2
Miscellaneous	14	520	4.4
Total	336	11 763	100.0

**Table 2:** Nutrients included in the eight nutrient profile models

*Model*	*Kcal*	*Added sugar*	*Total sugar*	*Fat*	*Saturated fat*	*Trans fat*	*Cholesterol*	*Sodium*	*Protein*	*Fibre*	*Fruit and veg*	*Vitamins/ Minerals*	*Total nutrients*	*Food Categories*
Brazilian			Y		Y	Y		Y					4	2
Danish			Y	Y				a					2	10
Disney	Y	Y	Y		Y	Y		Y					6	17
PepsiCo	Y	Y		Y	Y	Y	Y	Y	Y	Y		Y	9	7
UK	Y		Y		Y			Y	Y	Y	Y		7	2
US (Interagency)		Y			Y	Y		Y			Y		5	2
US (CSPI)		Y		Y		Y		Y					4	6
EUPNC	Y		Y	Y	Y			Y	Y	Y	Y	Y	9	20
Total	4	5	5	4	6	5	1	7	3	3	3	2		

a Note that the Danish model has a sodium criterion that is listed as ‘further consideration', but it is unclear when these further considerations should be taken into account.

**Table 3:** The number and percentage of foods and commercials classified as being suitable for advertising to children according to the selected models

*Model*	*Foods Approved*	*% Foods Approved (95% CIs)*	*% Commercials approved (95% CIs)*^a^
Brazilian	129	38.4 (33.2, 43.6)	44.01 (38.8, 49.2)
Danish^b^	124	36.9 (31.7, 42.1)	32.5 (27.4, 37.7)
Disney	8	2.4 (0.7, 4.0)	2.1 (0.4, 3.7)
PepsiCo	48	14.29 (10.5, 18.0)	14.4 (10.6, 18.1)
UK	134	39.88 (34.6, 45.1)	47.4 (42.1, 52.6)
US (CSPI)	70	20.8 (16.5, 25.2)	21.6 (17.3, 25.9)
US (Interagency)	46	13.7 (10.0, 17.4)	13.5 (9.8, 17.2)
EUPNC	86	25.6 (20.9, 30.3)	32.0 (27.3, 36.6)

a Note that standard errors are based on foods, rather than commercials, as repeated commercials do not introduce variance into the data set.

b Note that when the optional sodium criterion is applied the % of foods approved by the Danish model is 13.4% and the % of commercials approved is 16.14%.

**Table 4:** Pairwise kappa values (showing level of agreement adjusted for that expected by chance; *=‘fair', **=‘moderate', ***=‘good'^25^), weighted by number of commercials

	*Danish*	*Disney*	*PepsiCo*	*UK*	*US (CSPI)*	*US (Interagency)*	*EUPNC*
Brazilian	0.46**	0.05	0.12	0.73***	0.11	0.10	0.31*
Danish		0.04	0.12	0.49**	0.19	0.10	0.21*
Disney			0.12	0.04	0.08	0.13	0.06
PepsiCo				0.19	0.65***	0.65***	0.30*
UK					0.23*	0.22*	0.39*
US (CSPI)						0.52**	0.37*
US (Interagency)							0.36*

## Box 1 The eight nutrient profile models included in the analysis

*Brazilian model:*^15^ Developed in 2006 by the National Health Surveillance Agency (Anvisa), a government organisation, and introduced in 2010 to regulate food advertising to both adults and children. The model is based on negative nutrients only (that is, nutrients that are associated with adverse health outcomes).

*Danish model:*^16^ Developed by the Forum of Responsible Food Marketing Communication and introduced in 2008 as part of a voluntary code for food advertising. The Forum is a cooperation between nine commercial associations and the Danish Chamber of Commerce. The model is based on negative nutrients only.

*Disney model:*^17^ Developed by nutrition professionals for the Disney corporation and introduced in 2007 (updated in 2012) to regulate food advertising during programmes targeting children and families. The current version of the model is predominantly based on negative nutrients, but some food categories include a measure of positive food groups (for example, fruit and vegetables, whole grains).

*PepsiCo model:*^18^ Developed by PepsiCo in 2005. The model is used to regulate advertising of PepsiCo products with a target audience under 12 years of age. The model is based on negative and positive nutrients and food components.

*UK model:*^19^ Developed in 2005 by the Food Standards Agency, a government agency. Since 2007 the model has been used to support the regulation of food advertising during programmes aimed at children under the age of 16. The model is based on both negative and positive nutrients and food components. External validity of the model has been demonstrated.^20, 21^

*US (CSPI) model:*^22^ Developed by the Center for Science and the Public Interest (CSPI), a non-government organisation, and proposed as a set of voluntary guidelines for food manufacturers and media outlets to regulate advertising of foods to children in 2005. The model is based on negative nutrients, with a criterion that foods must contain at least 10% of the daily recommended intake of a positive nutrient, or contain half a serving of a fruit or vegetable, or be more than 51% wholegrain.

*US (Interagency) model:*^23^ Developed by the Interagency Working Group on Food Marketed to Children, a collaboration between the Federal Trade Commission, the Center for Disease Control and Prevention, the Food and Drug Administration, and the US Department of Agriculture in 2009. The model was developed to assist food manufacturers' determine which foods should be advertised to children. The model examines whether a food item provides a meaningful contribution to a healthy diet by considering nine positive food groups, and the model also uses negative nutrients.

*EU Pledge Nutrition Criteria:*^24^ The EU Pledge was launched as a voluntary initiative in 2007. Pledge signatories committed not to advertise unhealthy foods to children. The EU Pledge Nutrition Criteria were developed in November 2012 to replace individual nutrient profiling models formulated by EU Pledge signatories. The model uses both positive and negative nutrients and food components.

## Notes

PS, CP, CA, AK, AM and MR were funded by the British Heart Foundation and have no conflicts of interest to declare. JH and EB were funded by the University of Liverpool; the research group that JH and EB work for has accepted funding from the California Prunes Board, Danone and Coca-Cola, but neither JH nor EB take personal consultancy and own no stock.
