# Supplementary material for: How important is the choice of the nutrient profile model used to regulate broadcast advertising of foods to children? A comparison using a targeted data set
Source: Eur J Clin Nutr. 2013 Jun 26;67(8):815–20. doi: 10.1038/ejcn.2013.112 (PMC3736515; doi:10.1038/ejcn.2013.112)
Supplement: Supplementary Materials 1 [file ejcn2013112x1.doc]

Supplementary Material 1: Foods included and their categorisations according to the UK Food Guide

Please note: the foods that appear more than once do so because they were advertised as an individual product, and then were subsequently the food that was randomly selected to represent a product range advertised in a different commercial.

| Bread, Cereals and Potatoes |
| --- |
| Albert Bartlett Rooster Potatoes |
| Big Bear t/a/ Honey Monster Foods Sugar Puffs |
| Cocorocks Breakfast Cereal |
| Giovanni Rana Fresh Pasta Cappeleti Ham and Cheese |
| Hovis Best of Both Bread |
| Hovis Wholemeal Bread |
| Kellogg’s Cornflakes |
| Kellogg’s Cornflakes Breakfast Cereal with Honey |
| Kellogg’s Crunchy Nut |
| Kellogg’s Frosties |
| Kellogg’s Special K Oats and Honey |
| Kellogg's Bran Flakes |
| Kellogg's Cocopops |
| Kellogg's Cocopops Moons and Stars |
| Kellogg’s Crunchy Nut Bites |
| Kellogg’s Multigrain Crunchy Nut Cornflakes |
| Kellogg's Optivia Berry Oat Crisp |
| Kellogg's Optivita Raisin Oat Crisp |
| Kellogg's Rice Krispies |
| Kellogg’s Special K Bliss |
| Kellogg’s Special K Bliss |
| Kellogg's Special K Sustain Creamy Berry Crunch |
| Kingsmill 50/50 Bread with Omega 3 |
| Kingsmill Everyday Loaf |
| Kingsmill Gold Lightly Seeded One |
| McCain Homefries |
| McCain Potato Gourmet Frozen Potato Rosti |
| McCain Rustic Oven Chips |
| Mission Deli Tortilla Wraps Original Flavour |
| Mission Orange Breakfast Loaf |
| Morrison's Supermarket advertising Kellogg’s Special K |
| Nestle Bitesize Shredded Wheat |
| Nestle Cheerios |
| Nestle Nesquik |
| Nestle Raisin Oats and More |
| Nestle Shredded Wheat |
| Nestle Shreddies |
| New York Bagels Original Flavour |
| Nimble White Lower Calorie Bread |
| Quaker Oats Porridge |
| Ready Brek Porridge |
| Tilda Microwaveable Rice |
| Uncle Ben’s Microwaveable Rice |
| Warburton's Seeded Batch |
| Warburton's White Bread |
| Weetabix |
| Weetabix Bitesize Minis with Honey |
| Weetabix Oatibix |
| Weetabix Weetos |
| Weetos Chocolate Hoop Cereal |
| Yorkshire Tea Cakes: Marmalade Cake |
|  |
| Composite Foods |
| Bachelor's Soupfulls Tomato and Basil Flavour |
| Baxter’s Cream of Chicken Soup |
| BirdsEye Eat Positive Traditional Turkey Dinner Frozen Ready Meal |
| Blue Dragon Sauces Hoi Sin Sauce |
| Branstons Burger Relish |
| Burger King 3 Pepper Angus |
| Burger King 3 Pepper Angus Burger |
| Burger King 6 pack Angus |
| Burger King Angus Burger with BBQ Sauce |
| Burger King Class Angus Burger |
| Burger King Meat Beast Burger |
| Burger King Whopper with Cheese |
| Dolmio Bolognese Cook In Sauce |
| Dolmio Taste of Italy Tomato, Garlic and Basil Sauce |
| Domino's Large Hot n Spicy Pizza |
| Domino's Mighty Meaty Regular Crust Medium Pizza |
| Domino's Mighty Meaty Regular Crust Medium Takeaway Pizza |
| Dr Oetker Mozzarella Topped Frozen Pizza |
| Ella's Kitchen Organic Tomato Based Pasta Sauce |
| Feasters Microwaveable Chargrilled Chicken Burger |
| Findus Lasagne Novelli |
| Findus Lemon and Honey Roast Chicken Frozen Meal |
| Ginsters of Cornwall Chicken Balti Pasty |
| Giovanni Rana Fresh Ricotta and Spinach Pasta |
| Goodfellas BBQ Chicken Pizza |
| Goodfellas Signature Five Cheese Frozen Pizza |
| Greggs the Bakers Mexican Chicken Oval Bite |
| Greggs the Bakers Steak Bake |
| Heinz Farmers Market Plum Tomato and Basil Flavour Soup |
| Heinz Spread and Bake Toast Toppers: Mushroom and Bacon |
| Heinz Weight Watchers Ready Meals Salmon and Broccoli with Potato Wedges and Creamy Sauce |
| KFC Bargain Bucket (16pc bucket with 6 fries) |
| KFC Boneless Banquet for One |
| KFC Fully Loaded Box Meal |
| Lloyd Grossman Madras Curry Sauce |
| Masterfoods Uncle Bens Chicken and Mushroom Risotto |
| Masterfoods Uncle Bens Express Egg Fried Rice |
| McDonalds Big Mac Meal |
| McDonalds Breakfast Sausage Egg and Cheese Bagel |
| McDonalds California Classic Burger |
| McDonalds Chicken Deluxe with Bacon |
| McDonalds Chicken Legend with Bacon |
| McDonalds Chicken Legend with Mayo |
| McDonalds Double Cheeseburger |
| McDonalds Happy Meal (chicken nuggets, bottled water, carrot sticks) |
| McDonalds Happy Meal (chicken nuggets, bottled water, fruit bag) |
| McDonalds Happy Meal (chicken nuggets, carrot sticks, small Fanta) |
| McDonalds Happy Meal (chicken nuggets, organic milk, carrot sticks) |
| McDonalds Happy Meal (chicken nuggets, organic milk, fruit bag) |
| McDonalds Happy Meal (fish fingers, bottled water, carrot sticks) |
| McDonalds Happy Meal (fish fingers, organic milk, fruit bag) |
| McDonalds Happy Meal (hamburger, bottled water, carrot sticks) |
| McDonalds Happy Meal (hamburger, bottled water, carrot sticks) |
| McDonalds Hot n Spicy Sweet Chilli Wrap |
| McDonalds M Burger |
| McDonalds Miami Melt |
| McDonalds New York Supreme |
| McDonalds Summer Menu Chicken Selects |
| Muller Rice Vanilla Custard Flavour Creamy Rice Pudding |
| Oakhouse Foods Shanghai Beef |
| Old El Paso Enchiladas Dinner Kit |
| Old El Paso Fajita Dinner Kit Original Smoky BBQ Fajitas |
| Old El Paso Stand and Stuff Taco Dinner Kit |
| Pizza Hut Double Pepperoni Large Stuffed Crust 14 inch Pizza |
| Pizza Hut Macaroni and Cheese |
| Pizza Hut Takeaway Medium Italian Meat Feast Pizza |
| Pizza Hut Veggie Supreme Regular Pan 9 inch |
| Pot Noodle Chow Mein Flavour |
| Rustlers Flame Grilled The Big One Burger |
| Sacla Pesto Sauce |
| Schwartz Indian Chicken Biryani Recipe Mix |
| Subway BBQ Rib Sub |
| Subway Chicken and Bacon Ranch Sub |
| Subway Meatball Marinara Sub |
| Subway Veggie Delux Sub |
| Uncle Ben's Microwaveable Risotto Bacon and Mushroom |
| Uncle Ben's Original Sweet and Sour Stir Fry Sauce |
| Unilever Golden Wonder Pot Noodle Doner Kebab Flavour |
| Weight Watchers Tuna in Thousand Island Dressing |
| Wiltshire Farm Foods Omelette, Chips and Beans |
|  |
| Fatty and Sugary Foods |
| Anchor Butter |
| Anchor Lighter Spreadable Butter |
| Aunt Bessie’s Yorkshire Puddings |
| Bahlsen Milk Chocolate Leibniz Biscuits |
| Bertolli Butter (Light) |
| Betty Crocker Chocolate Fudge Brownie Mix |
| Cadbury’s Chocolate Digestives |
| Cadbury’s Cream Egg Twisted |
| Cadbury's Crème Egg |
| Cadbury's Crunchie Bar |
| Cadbury's Dairy Milk Chocolate |
| Cadbury's Flake |
| Carte D'Or Strawberry and Yoghurt Delice Ice Cream |
| Clover Butter |
| Coca Cola Soft Drink |
| Coke Zero Soft Drink |
| Countrylife Butter |
| Crusha Strawberry Flavour Squash |
| Diet Coke Soft Drink |
| Dr Oetker Cake Decorations Coloured Ready to Roll Icing |
| Dr Pepper Soft Drink |
| Fanta Orange Flavoured Soft Drink |
| Fanta Still Orange Flavoured Soft Drink |
| Feel Good Drinks Juice Drinks |
| Ferrero Nutella Hazelnut Chocolate Spread |
| Ferrero Rocher Whole Roasted Hazelnut Encased in Thin Wafer Shell |
| Flora Buttery Margerine |
| Flora Margerine |
| Flora Pro-Activ Margarine-type Spread |
| Fox's Golden Crunch Biscuits |
| Fox's Melts Butter Crinkles Biscuits |
| Fruitella 2 Fruity Fruit Sweets |
| Fruitella Crunchies |
| Galaxy Chocolate |
| Galaxy Minstrels Chocolate |
| Galaxy Revels Chocolates |
| Galaxy Ripple Chocolate Bar |
| Go Ahead Bar Blueberry Flavour |
| Haagen-Dazs Chocolate Chip Ice Cream |
| Haribo Maoam Stripes Chewy Fruit Flavour Sweets |
| Haribo Starmix Jelly Sweets |
| Horlicks Extra Light Malted Milk Hot Drink |
| I Can't Believe It's Not Butter Margerine |
| Juicy Drop Pop Lollipop |
| Jus Rol Puff Pastry |
| Kellogg's Nutrigrain Cereal Bar Soft Bake Apple Flavour |
| Kellogg's Nutrigrain Soft Oaties Elevensies Chocolate Chip |
| Kelloggs Rice Krispies Squares Chocolate and Caramel Flavour |
| Kellogg's Special K Mini Breaks Caramel Flavour |
| Kellogg's Special K Snack Bars Red Berry |
| Kerrygold Soft Butter |
| Kettle Chips Lightly Salted Handcooked Crisps |
| Kinder Bueno |
| Kinder Bueno White |
| Kraft Oreo Biscuits |
| Lindt Lindor Chocolate Balls |
| Lindt Lindor Gold Chocolate Bunny |
| Lucozade Sport Orange Flavoured Isotonic Drink |
| Lurpak Butter |
| Lurpak Reduced Fat Butter |
| Mars Bar Chocolate |
| Mars Celebrations Chocolate Selection |
| Mars Maltesers |
| Mars Planets Chocolate Balls |
| Mars Skittles Original Fruit Sweets |
| Maryland Chocolate Chip Cookies |
| Maynard’s Wine Gums Chewy Firm Sweets |
| McCoy’s Flame Grilled Steak Flavour Crisps |
| McDonalds Crunchie McFlurry |
| McDonalds Dairy Milk McFlurry Ice-cream |
| McVitie's Chocolate Digestives |
| McVitie's Jaffa Cakes |
| McVitie's Plain Digestives |
| Mr Kipling Cake Bites French Fancies |
| Mr Kipling Manor House Cake |
| Nature Valley Granola Maple Syrup Flavour |
| Nestle Aero Bubbles Chocolate Balls |
| Nestle Aero Chocolate bar |
| Nestle Aero Hot Chocolate |
| Nestle Kit Kat |
| Nestle Kit Kat Senses |
| Nestle Milkybar White Chocolate Bar |
| Nestle Quality Street Chocolate Selection |
| Nestle Rowntrees Fruit Pastilles Tube |
| Nestle Smarties |
| Oasis Fruit Squash Citrus Punch |
| Ocean Spray Cranberry Juice |
| Orangina Orange Soft Drink |
| Pepperami Pork Sausage Snack |
| Pepsi Max Soft Drink |
| Perfetti Van Melle Mentos Rainbow Flavour Mints |
| Philadelphia Splendips |
| Pom-Bear Bear Shaped Potato Crisps Original |
| President Butter Unsalted |
| Pringles Rice Infusions Cheese and Chilli Flavour Rice Crisps |
| Pringles Salt and Pepper Flavour Crisps |
| Pringles Select Multigrain Crisps |
| Robinson's Fruit Shoot H20 Apple Flavour |
| Robinson's Fruit Shoot Juice Blackcurrant Flavour |
| Robinson’s Orange Fruit Squash |
| Rubicon Lychee Fruit Juice Drinks |
| Ryvita Limbos Baked Crisps Cheese and Chive |
| Ryvita Mini's Salt & Vinegar Flavour Rye Snacks |
| Ryvita Mini's Sweet Chilli Flavour Rye Snacks |
| Schweppes Tonic Water |
| Sliderz Lollipops |
| Snack-a-jacks Ready Salted Flavour Rice and Corn Snacks |
| Snickers Chocolate and Peanut Bar |
| Starburst Choozers Chewy Fruit Flavoured Sweets |
| Starburst Fruit Sweets |
| Terry's Chocolate Orange |
| The Natural Confectionery Co. Jelly Snakes |
| Tic Tacs |
| Tic Tacs Orange Mints |
| Toblerone Chocolate |
| Turnocks Teacakes |
| Twix Chocolate Bar |
| Unilever Golden Magnum |
| Unilever Magnum Classic |
| Unilever Magnum Mayan Mystica |
| Unilever Magnum Mini Chocolate Covered Ice Cream |
| Vimto Purple Soft Drink |
| Walker’s Sun Bites Sour Cream Flavour |
| Walker’s Baked Ready Salted Flavour Potato Crisps |
| Walker’s Cheese and Onion Crisps |
|  |
| Fruit and Vegetables |
| BirdsEye Frozen Garden Peas |
| Campbells V8 Vegetable Juice |
| Ella's Kitchen Green Organic Fruit Smoothie |
| Florette Lambs Lettuce and Ruby Chard Bag of Salad |
| Green Giant Tinned Sweetcorn |
| Heinz Beanz Baked Beans Snap Pots |
| Innocent Pomegranate, Acai and Blueberry Smoothie |
| Tropicana Orange Juice |
| Tropicana Tropical Fruit Fury Smoothie |
| Welch's Purple Grape Juice |
|  |
| Meat, Fish and Alternatives |
| Bernard Matthews Breaded Turkey Drummers |
| BirdsEye Cheddar Cheese and Brocolli Meal |
| BirdsEye Crispy Chicken Breasts |
| BirdsEye Fishfingers |
| BirdsEye Fishfingers |
| Goldenlay Omega 3 Free Range Eggs |
| Hormel Foods Spam |
| Hormel Foods Spam Fritters |
| Mattessons Fridge Raiders Bites Roast Chicken Pieces |
| Mattessons Smoked Pork Sausage |
| Quorn Low Fat Chicken Style Pieces |
| Quorn Mince Meat Substitute |
| Walls Sausages |
| Young’s Fish Fillet Pollock Fish Pie |
| Young's Great Grimsby Cod Fillets in Breadcrumbs |
|  |
| Milk and Dairy |
| Alpro Soya Strawberry and Banana Dairy Free Yoghurt Alternative |
| Apetina Classic Block Feta Cheese |
| Babybel Cheese in Red Wax Casing |
| Ben & Jerrys Low Fat Frozen Yoghurt Chocolate Fudge Brownie Flavour |
| Benecol Light Low Fat Yoghurt Drink |
| Cathedral City Lighter Mature Cheddar |
| Cathedral City Mature Cheddar |
| Cheestrings Original Processed Cheese Strips |
| Cravendale Semi-Skimmed Milk |
| Dairylea Cheese Slices |
| Dairylea Cheese Triangles |
| Dairylea Dunkers Cheese with Dipping Sticks |
| Dairylea Dunkers Nachos |
| Danone Actimel Probiotic Drinking Yoghurt Raspberry |
| Danone Activia Fat Free Probiotic Yoghurt |
| Danone Activia Intensely Creamy Cherry Flavour Yoghurt |
| Danone Activia Prune Flavour Probiotic Yoghurt |
| Danone Shape Peach and Passion Fruit Yoghurt |
| Dr.Oetker Onken Strawberry Wholegrain Yoghurt |
| Galbani St Lucia Mozzarella |
| Hellman's Light Mayonnaise |
| Hellman’s Squeezy Light Mayonnaise |
| Lactofree Semi Skimmed Lactose Free Milk |
| Laughing Cow Cheese Spread |
| Le Roule Light Mild Cheese with Garlic and Herbs |
| Leerdammer Dutch Semi-Hard Cheese |
| McLelland Seriously Strong Cheddar Cheese |
| Muller 1 a Day Yoghurt Lemon Cheesecake |
| Muller Fruit Corner Peach and Apricot Yoghurt |
| Muller Light Fat Free Forest Fruit Flavour Yoghurt |
| Muller Little Stars Fromage Frais |
| Muller One-A-Day Yoghurt Based Smoothie |
| Nestle Munch Bunch Fromage Frais Pots |
| Philadelphia Garlic and Herb Cream Cheese |
| Philadelphia Light Soft Cheese |
| Philadelphia Soft Cheese |
| President Emmenthal Medium Hard Cheese |
| Primula Cheese |
| Wykefarm’s Extra Mature Cheese |
| Yakult Probiotic Yoghurt Drink |
| Yoplait Dairy Crest Petit Filous |
| Yoplait Dairy Crest Petit Filous Frubes Halloween Edition |
|  |
| Miscellaneous |
| Blue Dragon Sweet Chilli Dipping Sauce |
| Buxton Bottled Water |
| Colman's Pork Casserole Recipe Mix |
| Discovery Mild Fajita Seasoning Mix |
| Drench Bottled Water |
| Gourmet Garden Basil Paste |
| Heinz Roasted Garlic Deli Mayo |
| Heinz Salad Cream |
| Hellman's Real Mayonnaise |
| Isklar Mineral Water |
| Knorr Chicken Stock Pot |
| Marmite Yeast Extract Spread |
| Seven Seas Multibionta Multivitamins |
