# Supplementary material for: How important is the choice of the nutrient profile model used to regulate broadcast advertising of foods to children? A comparison using a targeted data set
Source: Eur J Clin Nutr. 2013 Jun 26;67(8):815–20. doi: 10.1038/ejcn.2013.112 (PMC3736515; doi:10.1038/ejcn.2013.112)
Supplement: Supplementary Data [file ejcn2013112x2.doc]

*** List of Variables Used

* Variables generated in the course of individual models' .do files are not listed

* ad_frequency : number of adverts

* biotin : biotin UNITS

* brand_name : brand name of advertised food

* brazilian_exempt : whether food is exempt from the Brazilian model (1=exempt, 0=not exempt)

* calcium_mg : calcium (mg/100g)

* cholesterol : cholesterol (mg/100g)

* cis_mon_fa : monounsaturated fatty acids (g/100g)

* cis_n3_fa : cis-n3 unsaturated fatty acids (g/100g)

* cis_n6_fa : cis-n6 unsaturated fatty acids (g/100g)

* danish_cat : food category according to the Danish model

* disney_approved_food_count : number of approved food groups the food has according to the Disney model

* disney_cat : food category according to the Disney model

* energy_kcal : energy (kcal/100g)

* energy_kj : energy (kJ/10g)

* eupledge_category : food category according to the EU Pledge model

* fat_g : fat (g/100g)

* fattysugary : if the food is in the "Fatty and Sugary Foods" category, this variable states whether it is a snack, non-snack, or drink item

* fibre_g : fibre (g/100g)

* folate : folate(µg/100g)

* food_category : UK Food Guide Category

* food_description : description of the advertised food

* food_to_encourage : whether food contains a 'food group to encourage' as defined by the PepsiCo model (1=yes, 0=no)

* fvhalf : whether food contains half a portion of fruit/veg (as required for some categories in the EU Pledge model)(0=yes, 1=no)

* interagency_combination_percent : percentage of foods to encourage, as defined in the US Interagency model

* iron_mg : iron (mg/100g)

* magnesium_mg : magnesium (mg/100g)

* mealcomponentpass : whether meal contains a component to encourage according to the criteria set out in the EU Pledge model (0=yes, 1=no)

* mealtype : whether meal is a breakfast or lunch meal, used for the EU Pledge model ('breakfast' or 'lunch')

* niacin : niacin (mg/100g)

* non_milk_extrinsic_sugars : non-milk extrinsic sugars (g/100g)

* pantothenic_acid : pantothenic acid (mg/100g)

* pepsico_cat : food cateogory according to the PepsiCo model

* potassium_mg : potassium (mg/100g)

* protein_g : protein (g/100g)

* reference_quantity : reference quantity used in the PepsiCo model

* riboflavin : riboflavin (mg/100g)

* saturates_g : saturated fat (g/100g)

* serving_size_g : serving size (g)

* sodium_mg : sodium (mg/100g)

* thiamin : thiamin (mg/100g)

* total_sugars_g : total sugars (g/100g)

* trans_fa : trans fatty acids (g/100g)

* vitamin_a : vitamin A (µg/100g)

* vitamin_b12 : vitamin B12 (µg/100g)

* vitamin_b6 : vitamin B6 (µg/100g)

* vitamin_c : vitamin C (mg/100g)

* vitamin_d : vitamin D (µg/100g)

* vitamin_e : vitamin E (mg/100g)

* wxyfm_cat : food category according to the WXYfm model

* wxyfm_fvn_percentage : total percentage of fruit, vegetables, and nuts

* zinc_mg : zinc (mg/100g)

*UK WXYfm model*

*Categories:

* "Food"

* "Drink"

**Energy score**

generate energy_score = energy_kj

replace energy_score = 0 if (energy_kj <= 335)

replace energy_score = 1 if (energy_kj > 335) & (energy_kj <=670)

replace energy_score = 2 if (energy_kj > 670) & (energy_kj <= 1005)

replace energy_score = 3 if (energy_kj > 1005) & (energy_kj <= 1340)

replace energy_score = 4 if (energy_kj > 1340) & (energy_kj <= 1675)

replace energy_score = 5 if (energy_kj > 1675) & (energy_kj <= 2010)

replace energy_score = 6 if (energy_kj > 2010) & (energy_kj <= 2345)

replace energy_score = 7 if (energy_kj > 2345) & (energy_kj <= 2680)

replace energy_score = 8 if (energy_kj > 2680) & (energy_kj <= 3015)

replace energy_score = 9 if (energy_kj > 3015) & (energy_kj <= 3350)

replace energy_score = 10 if (energy_kj > 3350)

**Saturated Fat score**

generate Sat_fat_score = saturates_g

replace Sat_fat_score = 0 if (saturates_g <= 1)

replace Sat_fat_score = 1 if (saturates_g > 1) & (saturates_g <= 2)

replace Sat_fat_score = 2 if (saturates_g > 2) & (saturates_g <= 3)

replace Sat_fat_score = 3 if (saturates_g > 3) & (saturates_g <= 4)

replace Sat_fat_score = 4 if (saturates_g > 4) & (saturates_g <= 5)

replace Sat_fat_score = 5 if (saturates_g> 5) & (saturates_g <= 6)

replace Sat_fat_score = 6 if (saturates_g > 6) & (saturates_g <= 7)

replace Sat_fat_score = 7 if (saturates_g > 7) & (saturates_g <= 8)

replace Sat_fat_score = 8 if (saturates_g > 8) & (saturates_g <= 9)

replace Sat_fat_score = 9 if (saturates_g > 9) & (saturates_g <= 10)

replace Sat_fat_score = 10 if (saturates_g > 10)

**Total sugars Score**

generate sugars_score = total_sugars_g

replace sugars_score = 0 if (total_sugars_g <= 4.5 )

replace sugars_score = 1 if (total_sugars_g > 4.5) & (total_sugars_g <= 9)

replace sugars_score = 2 if (total_sugars_g > 9) & (total_sugars_g <= 13.5)

replace sugars_score = 3 if (total_sugars_g > 13.5) & (total_sugars_g <= 18)

replace sugars_score = 4 if (total_sugars_g > 18) & (total_sugars_g <= 22.5)

replace sugars_score = 5 if (total_sugars_g > 22.5) & (total_sugars_g <= 27)

replace sugars_score = 6 if (total_sugars_g > 27) & (total_sugars_g <= 31)

replace sugars_score = 7 if (total_sugars_g > 31) & (total_sugars_g <= 36)

replace sugars_score = 8 if (total_sugars_g > 36) & (total_sugars_g <= 40)

replace sugars_score = 9 if (total_sugars_g > 40) & (total_sugars_g <= 45)

replace sugars_score = 10 if (total_sugars_g > 45)

**Sodium score**

generate S_mg = sodium_mg

generate sodium_score = S_mg

replace sodium_score = 0 if (S_mg <= 90)

replace sodium_score = 1 if (S_mg > 90) & (S_mg <= 180)

replace sodium_score = 2 if (S_mg > 180) & (S_mg <= 270)

replace sodium_score = 3 if (S_mg > 270) & (S_mg <= 360)

replace sodium_score = 4 if (S_mg > 360) & (S_mg <= 450)

replace sodium_score = 5 if (S_mg > 450) & (S_mg <= 540)

replace sodium_score = 6 if (S_mg > 540) & (S_mg <= 630)

replace sodium_score = 7 if (S_mg > 630) & (S_mg <= 720)

replace sodium_score = 8 if (S_mg > 720) & (S_mg <= 810)

replace sodium_score = 9 if (S_mg > 810) & (S_mg <= 900)

replace sodium_score = 10 if (S_mg > 900)

**Fruit and Veg score**

generate wxyfm_FVN_score = wxyfm_fvn_percentage

replace wxyfm_FVN_score = 0 if (wxyfm_fvn_percentage <= 40)

replace wxyfm_FVN_score = 1 if (wxyfm_fvn_percentage > 40) & (wxyfm_fvn_percentage <= 60)

replace wxyfm_FVN_score = 2 if (wxyfm_fvn_percentage > 60) & (wxyfm_fvn_percentage <= 80)

replace wxyfm_FVN_score = 5 if (wxyfm_fvn_percentage > 80)

**Fibre Score**

generate fibre_score = fibre_g

replace fibre_score = 0 if (fibre_g <= 0.7)

replace fibre_score = 1 if (fibre_g > 0.7) & (fibre_g <= 1.4)

replace fibre_score = 2 if (fibre_g > 1.4) & (fibre_g <= 2.1)

replace fibre_score = 3 if (fibre_g > 2.1) & (fibre_g <= 2.8)

replace fibre_score = 4 if (fibre_g > 2.8) & (fibre_g <= 3.5)

replace fibre_score = 5 if (fibre_g > 3.5)

**Protein Score**

generate protein_score = protein_g

replace protein_score = 0 if (protein_g <= 1.6)

replace protein_score = 1 if (protein_g > 1.6) & (protein_g <= 3.2)

replace protein_score = 2 if (protein_g > 3.2) & (protein_g <= 4.8)

replace protein_score = 3 if (protein_g > 4.8) & (protein_g <= 6.4)

replace protein_score = 4 if (protein_g > 6.4) & (protein_g <= 8.0)

replace protein_score = 5 if (protein_g > 8.0)

**A Points and C Points**

generate A_points = energy_score + Sat_fat_score + sugars_score + sodium_score

generate C_points = wxyfm_FVN_score + fibre_score + protein_score

**Protein Cap Step 1**

generate protein_cap = 1 if (A_points >= 11) & (wxyfm_FVN_score < 5)

**Final score**

generate wxyfm_Score = A_points - C_points

**Protein cap step 2**

generate C_points_capped = fibre_score + wxyfm_FVN_score

generate Capped_wxyfm_score = A_points - C_points_capped

replace wxyfm_Score = Capped_wxyfm_score if protein_cap == 1

**WXYfm Pass/Fail**

generate wxyfm_classification = 1

replace wxyfm_classification = 0 if wxyfm_Score >= 4 & wxyfm_cat== "Food"

replace wxyfm_classification = 0 if wxyfm_Score >= 1 & wxyfm_cat== "Drink"

**Drop redundant variables**

drop energy_score Sat_fat_score sugars_score sodium_score wxyfm_FVN_score fibre_score protein_score S_mg

drop A_points C_points protein_cap C_points_capped Capped_wxyfm_score wxyfm_Score

** US Interagency model **

*Categories:

* Individual foods = 1

* Main dishes and meals = 2

generate interagency_category = 1

replace interagency_category = 2 if serving_size_g > 170

*Standard 1 >new name

*Exempt = 1

*Not exempt = 0

**Principle A**

*use Standard 1 column*

generate interagency_principle_a = 0

replace interagency_principle_a = 1 if (interagency_combination_percent >= 50)

*Principle B - Nutrients to limit*

*Sat Fat*

generate satfat_serving = (saturates_g/100)*serving_size_g

generate satfat_pc_calories = (( saturates_g*9)/energy_kcal)*100

generate interagency_sat_fat1 = 0

generate interagency_sat_fat2 = 0

*sat fat individual foods*

replace interagency_sat_fat1 = 1 if (satfat_serving <= 1) & (interagency_category == 1)

replace interagency_sat_fat2 = 1 if (satfat_pc_calories <= 15) & (interagency_category == 1)

*satfat main dishes and meals*

replace interagency_sat_fat1 = 1 if (saturates_g <= 1) & (interagency_category == 2)

replace interagency_sat_fat2 = 1 if (satfat_pc_calories <= 10) & (interagency_category == 2)

*satfat final*

generate interagency_final_satfat = 0

replace interagency_final_satfat = 1 if (interagency_sat_fat1 == 1) |(interagency_sat_fat2 == 1)

*Trans fat*

generate trans_serving = trans_fa / 100 * serving_size_g

generate interagency_trans_fat = .

replace interagency_trans_fat = 0 if trans_serving >= 0.5

replace interagency_trans_fat = 1 if trans_serving < 0.5

*added sugars > use NME sugars**

generate added_sugars_serving = (non_milk_extrinsic_sugars/100)*serving_size_g

generate added_sugars_pc_calories = ((non_milk_extrinsic_sugars*4)/energy_kcal)*100

generate interagency_added_sugars = 0

replace interagency_added_sugars = 1 if (added_sugars_serving <= 13) & (interagency_category == 1)

replace interagency_added_sugars = 1 if (non_milk_extrinsic_sugars <= 13) & (interagency_category == 2)

*sodium*

generate sodium_serving =(sodium_mg/100)*serving_size_g

generate interagency_sodium = 0

replace interagency_sodium = 1 if (sodium_serving <= 210) & (interagency_category == 1)

replace interagency_sodium = 1 if (sodium_serving <= 450) & (interagency_category == 2)

*score*

generate interagency_count = interagency_final_satfat + interagency_trans_fat + interagency_added_sugars + interagency_sodium + interagency_principle_a

generate interagency_final = 0

replace interagency_final = 1 if (interagency_standard_1 == 1)

replace interagency_final = 1 if (interagency_count == 5)

*labels*

generate interagency_classification = interagency_final

label define interagency_classification 1 "health_claim" 0 "no_claim"

label values interagency_classification interagency_classification

*drop redundant variables*

drop interagency_final interagency_trans_fat trans_serving interagency_count interagency_sodium sodium_serving interagency_added_sugars

drop added_sugars_pc_calories added_sugars_serving interagency_final_satfat interagency_sat_fat2

drop interagency_sat_fat1 satfat_pc_calories satfat_serving interagency_principle_a interagency_category

** CSPI model**

*Categories:

* 1 = low-nutrition beverage

* 2 = nutritious beverage

* 3 = snacks (chips crisps etc)

* 4 = cereals, soups, pasta, meats

* 5 = pizza, sandwiches

* 6 = meals

**nutrients**

generate fat_pc_cspi = ((fat_g*9)/ energy_kcal)*100

generate trans_sat_pc_cspi = (((trans_fa*9)/ energy_kcal)*100) + ((( saturates_g*9)/energy_kcal)*100)

generate sodium_serving_cspi = (sodium_mg/100)*serving_size_g

generate added_sugars_pc_cspi = ((non_milk_extrinsic_sugars*4)/energy_kcal)* serving_size_g

*Step 2*

generate fat_score = 0

replace fat_score = 1 if (fat_pc_cspi <= 35)

generate trans_sat_score = 0

replace trans_sat_score = 1 if ( trans_sat_pc_cspi <= 10)

generate added_sugars_score = 0

replace added_sugars_score = 1 if (added_sugars_pc_cspi <= 10)

generate sodium_score = 0

replace sodium_score = 1 if (cspi_cat == 3) & (sodium_serving_cspi <= 230)

replace sodium_score = 1 if (cspi_cat == 4) & (sodium_serving_cspi <= 480)

replace sodium_score = 1 if (cspi_cat == 5) & (sodium_serving_cspi <= 600)

replace sodium_score = 1 if (cspi_cat == 6) & (sodium_serving_cspi <= 770)

*categorisation*

generate cspi_score = fat_score + trans_sat_score + added_sugars_score + sodium_score

generate cspi_classification = 0

replace cspi_classification = 1 if (cspi_score == 4)

replace cspi_classification = 1 if (cspi_cat == 2)

replace cspi_classification = 0 if (cspi_cat == 0)

**Label**

label define cspi_classification 1 "high_quality" 0 "poor_quality"

label values cspi_classification cspi_classification

*Drop redundant variables*

drop cspi_score sodium_score added_sugars_score trans_sat_score fat_score

drop added_sugars_pc_cspi sodium_serving_cspi trans_sat_pc_cspi fat_pc_cspi

** PepsiCo model **

*Categories:

* 1 = savoury snack

* 2 = sweet snack

* 3 = cereals, sauces and dips

* 4 = Other foods

* 5 = ICBA-approved beverages

* 6 = Other beverages

* 7 = Seeds, nuts and nut butters

**nutrients**

generate fat_pc_pepsico = ((fat_g*9)/ energy_kcal)*100

generate sat_pc_pepsico = (( saturates_g*9)/energy_kcal)*100

generate trans_pepsico = (trans_fa/100)* reference_quantity

replace trans_pepsico = ((trans_fa/100)* serving_size_g) if missing(trans_pepsico)

generate cholesterol_pepsico = (cholesterol/100)* reference_quantity

replace cholesterol_pepsico = ((cholesterol/100)* serving_size_g) if missing(cholesterol_pepsico)

generate sodium_serving_pepsico = (sodium_mg/100)*reference_quantity

replace sodium_serving_pepsico = ((sodium_mg/100)* serving_size_g) if missing(sodium_serving_pepsico)

generate added_sugars_pc_pepsico = ((non_milk_extrinsic_sugars*4)/energy_kcal)*100

*Step 2*

generate fat_score = 0

replace fat_score = 1 if (fat_pc_pepsico <= 35)

replace fat_score = 1 if (fat_pc_pepsico <= 43) & (pepsico_cat == 1) & (food_to_encourage == 1)

generate sat_score = 0

replace sat_score = 1 if (sat_pc_pepsico <= 10)

replace sat_score = 1 if (pepsico_cat == 7) & (sat_pc_pepsico <= 15)

generate trans_score = 0

replace trans_score = 1 if ( trans_pepsico <= 0.5)

generate cholesterol_score = 0

replace cholesterol_score = 1 if (cholesterol_pepsico <= 30)

generate sodium_score = 0

replace sodium_score = 1 if (sodium_serving_pepsico <= 150)

generate added_sugars_score = 0

replace added_sugars_score = 1 if (added_sugars_pc_pepsico <= 10)

replace added_sugars_score = 1 if (added_sugars_pc_pepsico <= 25) & (pepsico_cat == 2) & (food_to_encourage == 1)

replace added_sugars_score = 1 if (added_sugars_pc_pepsico <= 25) & (pepsico_cat == 3) & (food_to_encourage == 1)

generate nutrient_to_encourage = 0

replace nutrient_to_encourage = 1 if (protein_g >= 0.1)

replace nutrient_to_encourage = 1 if (fibre_g >= 0.01 )

replace nutrient_to_encourage = 1 if (potassium_mg >= 0.01)

replace nutrient_to_encourage = 1 if (magnesium_mg >= 0.01)

replace nutrient_to_encourage = 1 if (iron_mg >= 0.01)

replace nutrient_to_encourage = 1 if (calcium_mg >= 0.01)

replace nutrient_to_encourage = 1 if (zinc_mg >= 0.01)

replace nutrient_to_encourage = 1 if (folate >= 0.01)

replace nutrient_to_encourage = 1 if (vitamin_a >= 0.01)

replace nutrient_to_encourage = 1 if (vitamin_d >= 0.01)

replace nutrient_to_encourage = 1 if (vitamin_c >= 0.01)

replace nutrient_to_encourage = 1 if (vitamin_e >= 0.01)

generate encouraged_score = 1

replace encouraged_score = 0 if (nutrient_to_encourage + food_to_encourage) == 0

*categorisation - initial criteria*

generate pepsico_score = fat_score + sat_score + trans_score + cholesterol_score + added_sugars_score + sodium_score + encouraged_score

generate pepsico_classification = 0

replace pepsico_classification = 1 if (pepsico_score == 7)

replace pepsico_classification = 1 if (pepsico_cat == 5)

replace pepsico_classification = 0 if (pepsico_cat == 6)

**snacks should be under 150 kcal per serving**

replace pepsico_classification = 0 if (pepsico_cat == 1) & ((energy_kcal/100)*reference_quantity >= 150)

replace pepsico_classification = 0 if (pepsico_cat == 2) & ((energy_kcal/100)*reference_quantity >= 150)

* Drop variables no longer required *

drop fat_pc_pepsico sat_pc_pepsico trans_pepsico cholesterol_pepsico sodium_serving_pepsico

drop added_sugars_pc_pepsico fat_score sat_score trans_score cholesterol_score sodium_score

drop added_sugars_score nutrient_to_encourage encouraged_score pepsico_score

* EU Pledge Nutrition Criteria *

*Categories described below

generate kcalpass = .

generate sodiumpass = .

generate satpass = .

generate sugarpass = .

generate componentpass = .

generate kcal_serving = energy_kcal / 100 * serving_size_g

generate satpercent = saturates_g * 9 / energy_kcal

generate proteinpercent = protein_g * 4 / energy_kcal

generate sodium_mg_serving = sodium_mg / 100 * serving_size_g

generate nme_sugars_serving = non_milk_extrinsic_sugars / 100 * serving_size_g

generate PUFApercent = (cis_n3_fa + cis_n6_fa) / fat_g

generate UFApercent = (cis_mon_fa + cis_n3_fa + cis_n6_fa) / fat_g

generate satpercentfat = saturates_g/fat_g*100

generate UFApercentenergy = (cis_mon_fa + cis_n3_fa + cis_n6_fa)* 9 / energy_kcal

generate micropass = 1

replace micropass = 0 if vitamin_d >= 0.75 | thiamin >= 0.165 | riboflavin >= 0.21 | niacin >= 2.4 | vitamin_b6 >= 0.21 | vitami_b12 >= 0.375 | folate >= 30 | pantothenic_acid >= 0.9 | calcium_mg >= 120 | biotin >= 7.5

replace micropass = 0 if wxyfm_cat == "Drinks" & (vitamin_d >= 0.375 | thiamin >= 0.0825 | riboflavin >= 0.105 | niacin >= 1.2 | vitamin_b6 >= 0.105 | vitami_b12 >= 0.1875 | folate >= 15 | pantothenic_acid >= 0.45 | calcium_mg >= 60 | biotin >= 3.75 )

* Category 1: Vegetable and Animal based oils, fats and fat containing spreads & emulsion-based sauces

*Sub-category A: Vegetable and Animal based oils, fats and fat containing spreads

replace kcalpass = 0 if eupledge_category == "1a" & kcal_serving <= 85

replace kcalpass = 1 if eupledge_category == "1a" & kcal_serving > 85

replace sodiumpass = 0 if eupledge_category == "1a" & sodium_mg <= 500

replace sodiumpass = 1 if eupledge_category == "1a" & sodium_mg > 500

replace satpass = 0 if eupledge_category == "1a" & satpercentfat <= 0.33

replace satpass = 1 if eupledge_category == "1a" & satpercentfat > 0.33

replace sugarpass = 0 if eupledge_category == "1a" & total_sugars_g <= 5

replace sugarpass = 1 if eupledge_category == "1a" & total_sugars_g > 5

replace componentpass = 0 if eupledge_category == "1a" & PUFApercent >= 0.25

replace componentpass = 1 if eupledge_category == "1a" & PUFApercent < 0.25

*Sub-category B: Emulsion-based sauces

replace kcalpass = 0 if eupledge_category == "1b" & kcal_serving <= 85

replace kcalpass = 1 if eupledge_category == "1b" & kcal_serving > 85

replace sodiumpass = 0 if eupledge_category == "1b" & sodium_mg <= 750

replace sodiumpass = 1 if eupledge_category == "1b" & sodium_mg > 750

replace satpass = 0 if eupledge_category == "1b" & satpercentfat <= 0.33

replace satpass = 1 if eupledge_category == "1b" & satpercentfat > 0.33

replace sugarpass = 0 if eupledge_category == "1b" & total_sugars_g <= 5

replace sugarpass = 1 if eupledge_category == "1b" & total_sugars_g > 5

replace componentpass = 0 if eupledge_category == "1b" & PUFApercent >= 0.25

replace componentpass = 1 if eupledge_category == "1b" & PUFApercent < 0.25

* Category 2: Fruits, vegetables and seeds, except oil

*Sub-category A: Products of fruits and vegetables except oils and potatoes

replace kcalpass = 0 if eupledge_category == "2a" & kcal_serving <= 170

replace kcalpass = 1 if eupledge_category == "2a" & kcal_serving > 170

replace sodiumpass = 0 if eupledge_category == "2a" & sodium_mg <= 300

replace sodiumpass = 1 if eupledge_category == "2a" & sodium_mg > 300

replace satpass = 0 if eupledge_category == "2a" & saturates_g <= 1.5

replace satpass = 1 if eupledge_category == "2a" & saturates_g > 1.5

replace sugarpass = 0 if eupledge_category == "2a" & total_sugars_g <= 15

replace sugarpass = 1 if eupledge_category == "2a" & total_sugars_g > 15

replace componentpass = 0 if eupledge_category == "2a" & fvhalf == 0

replace componentpass = 1 if eupledge_category == "2a" & fvhalf == 1

*Sub-category B: Potato and potato products, except dehydrated potato products

replace kcalpass = 0 if eupledge_category == "2b" & kcal_serving <= 170

replace kcalpass = 1 if eupledge_category == "2b" & kcal_serving > 170

replace sodiumpass = 0 if eupledge_category == "2b" & sodium_mg <= 300

replace sodiumpass = 1 if eupledge_category == "2b" & sodium_mg > 300

replace satpass = 0 if eupledge_category == "2b" & saturates_g <= 1.5

replace satpass = 1 if eupledge_category == "2b" & saturates_g > 1.5

replace sugarpass = 0 if eupledge_category == "2b" & total_sugars_g <= 5

replace sugarpass = 1 if eupledge_category == "2b" & total_sugars_g > 5

replace componentpass = 0 if eupledge_category == "2b"

*nutrients delivered through main ingredient not possible to calculate

*Sub-category C: Potato chips and potato based snack, including dough-based products

replace kcalpass = 0 if eupledge_category == "2c" & kcal_serving <= 170

replace kcalpass = 1 if eupledge_category == "2c" & kcal_serving > 170

replace sodiumpass = 0 if eupledge_category == "2c" & sodium_mg <= 670

replace sodiumpass = 1 if eupledge_category == "2c" & sodium_mg > 670

replace satpass = 0 if eupledge_category == "2c" & satpercent <= 10

replace satpass = 1 if eupledge_category == "2c" & satpercent > 10

replace sugarpass = 0 if eupledge_category == "2c" & total_sugars_g <= 10

replace sugarpass = 1 if eupledge_category == "2c" & total_sugars_g > 10

replace componentpass = 0 if eupledge_category == "2c" & (fibre_g >= 3 | UFApercent >= 0.7)

replace componentpass = 1 if eupledge_category == "2c" & (fibre_g < 3 & UFApercent < 0.7)

*Sub-category D: Seeds and Nuts (none in advertised foods database)*

*Sub-category E: Fruit/vegetable based meal sauces

replace kcalpass = 0 if eupledge_category == "2e" & kcal_serving <= 100

replace kcalpass = 1 if eupledge_category == "2e" & kcal_serving > 100

replace sodiumpass = 0 if eupledge_category == "2e" & sodium_mg <= 500

replace sodiumpass = 1 if eupledge_category == "2e" & sodium_mg > 500

replace satpass = 0 if eupledge_category == "2e" & saturates_g <= 1.5

replace satpass = 1 if eupledge_category == "2e" & saturates_g > 1.5

replace sugarpass = 0 if eupledge_category == "2e" & total_sugars_g <= 10

replace sugarpass = 1 if eupledge_category == "2e" & total_sugars_g > 10

replace componentpass = 0 if eupledge_category == "2e"

*componentpass includes nutrients delivered through ingredients

*Category 3: Meat based products

replace kcalpass = 0 if eupledge_category == "3" & kcal_serving <= 170

replace kcalpass = 1 if eupledge_category == "3" & kcal_serving > 170

replace sodiumpass = 0 if eupledge_category == "3" & sodium_mg <= 800

replace sodiumpass = 1 if eupledge_category == "3" & sodium_mg > 800

replace satpass = 0 if eupledge_category == "3" & saturates_g <= 6

replace satpass = 1 if eupledge_category == "3" & saturates_g > 6

replace sugarpass = 0 if eupledge_category == "3" & total_sugars_g <= 5

replace sugarpass = 1 if eupledge_category == "3" & total_sugars_g > 5

replace componentpass = 0 if eupledge_category == "3" & proteinpercent >= 0.12

replace componentpass = 1 if eupledge_category == "3" & proteinpercent < 0.12

*Category 4: Fishery products

replace kcalpass = 0 if eupledge_category == "4" & kcal_serving <= 170

replace kcalpass = 1 if eupledge_category == "4" & kcal_serving > 170

replace kcalpass = 0 if eupledge_category == "4" & kcal_serving > 170 & PUFApercent >=25

replace sodiumpass = 0 if eupledge_category == "4" & sodium_mg <= 450

replace sodiumpass = 1 if eupledge_category == "4" & sodium_mg > 450

replace satpass = 0 if eupledge_category == "4" & satpercent <= 0.33

replace satpass = 1 if eupledge_category == "4" & satpercent > 0.33

replace satpass = 0 if eupledge_category == "4" & satpercentfat <= 0.33

replace satpass = 1 if eupledge_category == "4" & satpercentfat > 0.33

replace sugarpass = 0 if eupledge_category == "4" & total_sugars_g <= 5

replace sugarpass = 1 if eupledge_category == "4" & total_sugars_g > 5

replace componentpass = 0 if eupledge_category == "4" & proteinpercent >= 0.12

replace componentpass = 1 if eupledge_category == "4" & proteinpercent < 0.12

*Category 5: Dairy products

*Sub-category A: Dairy products other than cheeses

replace kcalpass = 0 if eupledge_category == "5a" & kcal_serving <= 170

replace kcalpass = 1 if eupledge_category == "5a" & kcal_serving > 170

replace sodiumpass = 0 if eupledge_category == "5a" & sodium_mg <= 300

replace sodiumpass = 1 if eupledge_category == "5a" & sodium_mg > 300

replace satpass = 0 if eupledge_category == "5a" & saturates_g <= 2.6

replace satpass = 1 if eupledge_category == "5a" & saturates_g > 2.6

replace sugarpass = 0 if eupledge_category == "5a" & total_sugars_g <= 13.5

replace sugarpass = 1 if eupledge_category == "5a" & total_sugars_g > 13.5

replace componentpass = 1 if eupledge_category == "5a"

replace componentpass = 0 if eupledge_category == "5a" & (proteinpercent >= 0.12 | protein_g >= 2 | micropass == 0)

replace componentpass = 0 if eupledge_category == "5a" & (proteinpercent < 0.12 & protein_g < 2 & micropass == 1)

*Sub-category B: Cheese and savoury dairy based products

* i) Hard, semi-hard cheeses

replace kcalpass = 0 if eupledge_category == "5bi" & kcal_serving <= 85

replace kcalpass = 1 if eupledge_category == "5bi" & kcal_serving > 85

replace sodiumpass = 0 if eupledge_category == "5bi" & sodium_mg <= 900

replace sodiumpass = 1 if eupledge_category == "5bi" & sodium_mg > 900

replace satpass = 0 if eupledge_category == "5bi" & saturates_g <= 15

replace satpass = 1 if eupledge_category == "5bi" & saturates_g > 15

replace sugarpass = 0 if eupledge_category == "5bi" & total_sugars_g <= 5

replace sugarpass = 1 if eupledge_category == "5bi" & total_sugars_g > 5

replace componentpass = micropass if eupledge_category == "5bi"

* ii) Other cheeses, curd & quark and savoury dairy based products

replace kcalpass = 0 if eupledge_category == "5bii" & kcal_serving <= 170

replace kcalpass = 1 if eupledge_category == "5bii" & kcal_serving > 170

replace sodiumpass = 0 if eupledge_category == "5bii" & sodium_mg <= 800

replace sodiumpass = 1 if eupledge_category == "5bii" & sodium_mg > 800

replace satpass = 0 if eupledge_category == "5bii" & saturates_g <= 10

replace satpass = 1 if eupledge_category == "5bii" & saturates_g > 10

replace sugarpass = 0 if eupledge_category == "5bii" & total_sugars_g <= 8

replace sugarpass = 1 if eupledge_category == "5bii" & total_sugars_g > 8

replace componentpass = micropass if eupledge_category == "5bii"

* Category 6: Cereal based products

*Sub-category A: Sweet biscuits, fine bakery wares and other cereal based products

replace kcalpass = 0 if eupledge_category == "6a" & kcal_serving <= 200

replace kcalpass = 1 if eupledge_category == "6a" & kcal_serving > 200

replace sodiumpass = 0 if eupledge_category == "6a" & sodium_mg <= 450

replace sodiumpass = 1 if eupledge_category == "6a" & sodium_mg > 450

replace satpass = 0 if eupledge_category == "6a" & saturates_g <= 10

replace satpass = 1 if eupledge_category == "6a" & saturates_g > 10

replace sugarpass = 0 if eupledge_category == "6a" & total_sugars_g <= 35

replace sugarpass = 1 if eupledge_category == "6a" & total_sugars_g > 35

replace componentpass = 0 if eupledge_category == "6a" & (fibre_g >= 3 | UFApercent >= 0.7 | UFApercentenergy < 0.2)

replace componentpass = 1 if eupledge_category == "6a" & (fibre_g < 3 & UFApercent < 0.7 & UFApercentenergy < 0.2)

*whole grain stuff not possible to add due to lack of data

*Sub-category B: Savoury biscuits, fine bakery wares and other cereal based products, including dough based products

replace kcalpass = 0 if eupledge_category == "6b" & kcal_serving <= 170

replace kcalpass = 1 if eupledge_category == "6b" & kcal_serving > 170

replace sodiumpass = 0 if eupledge_category == "6b" & sodium_mg <= 900

replace sodiumpass = 1 if eupledge_category == "6b" & sodium_mg > 900

replace satpass = 0 if eupledge_category == "6b" & satpercent <= 0.1

replace satpass = 1 if eupledge_category == "6b" & satpercent > 0.1

replace sugarpass = 0 if eupledge_category == "6b" & total_sugars_g <= 10

replace sugarpass = 1 if eupledge_category == "6b" & total_sugars_g > 10

replace componentpass = 0 if eupledge_category == "6b" & (fibre_g >= 3 | UFApercent >= 0.7)

replace componentpass = 1 if eupledge_category == "6b" & (fibre_g < 3 & UFApercent < 0.7)

*Sub-category C: Breakfast cereals including porridge

replace kcalpass = 0 if eupledge_category == "6c" & kcal_serving <= 210

replace kcalpass = 1 if eupledge_category == "6c" & kcal_serving > 210

replace sodiumpass = 0 if eupledge_category == "6c" & sodium_mg <= 450

replace sodiumpass = 1 if eupledge_category == "6c" & sodium_mg > 450

replace satpass = 0 if eupledge_category == "6c" & saturates_g <= 5

replace satpass = 1 if eupledge_category == "6c" & saturates_g > 5

replace sugarpass = 0 if eupledge_category == "6c" & total_sugars_g <= 30

replace sugarpass = 1 if eupledge_category == "6c" & total_sugars_g > 30

replace componentpass = 0 if eupledge_category == "6c" & fibre_g >= 3

replace componentpass = 1 if eupledge_category == "6c" & fibre_g < 3

*whole grain stuff not possible to add due to lack of data

*Sub-category D: Cereal and cereal products except breakfast cereals, biscuits and fine bakey wares

replace kcalpass = 0 if eupledge_category == "6d" & kcal_serving <= 340

replace kcalpass = 1 if eupledge_category == "6d" & kcal_serving > 340

replace sodiumpass = 0 if eupledge_category == "6d" & sodium_mg <= 500

replace sodiumpass = 1 if eupledge_category == "6d" & sodium_mg > 500

replace satpass = 0 if eupledge_category == "6d" & saturates_g <= 5

replace satpass = 1 if eupledge_category == "6d" & saturates_g > 5

replace sugarpass = 0 if eupledge_category == "6d" & total_sugars_g <= 5

replace sugarpass = 1 if eupledge_category == "6d" & total_sugars_g > 5

replace componentpass = 0 if eupledge_category == "6d" & fibre_g >= 3

replace componentpass = 1 if eupledge_category == "6d" & fibre_g < 3

*whole grain stuff not possible to add due to lack of data

* Category 7: Soups, composite dishes, main course and filled sandwiches

*Sub-category A: Soups

replace kcalpass = 0 if eupledge_category == "7a" & kcal_serving <= 170

replace kcalpass = 1 if eupledge_category == "7a" & kcal_serving > 170

replace sodiumpass = 0 if eupledge_category == "7a" & sodium_mg <= 350

replace sodiumpass = 1 if eupledge_category == "7a" & sodium_mg > 350

replace satpass = 0 if eupledge_category == "7a" & saturates_g <= 1.5

replace satpass = 1 if eupledge_category == "7a" & saturates_g > 1.5

replace sugarpass = 0 if eupledge_category == "7a" & total_sugars_g <= 7.5

replace sugarpass = 1 if eupledge_category == "7a" & total_sugars_g > 7.5

replace componentpass = 0 if eupledge_category == "7a"

*not possible to determine whether nutrients delivered through main ingredient

*Sub-category B: Composite dishes, main dishes, and filled sandwiches

replace kcalpass = 0 if eupledge_category == "7b" & kcal_serving <= 425

replace kcalpass = 1 if eupledge_category == "7b" & kcal_serving > 425

replace sodiumpass = 0 if eupledge_category == "7b" & sodium_mg <= 400

replace sodiumpass = 1 if eupledge_category == "7b" & sodium_mg > 400

replace satpass = 0 if eupledge_category == "7b" & saturates_g <= 5

replace satpass = 1 if eupledge_category == "7b" & saturates_g > 5

replace sugarpass = 0 if eupledge_category == "7b" & total_sugars_g <= 7.5

replace sugarpass = 1 if eupledge_category == "7b" & total_sugars_g > 7.5

replace componentpass = 0 if eupledge_category == "7b"

*not possible to determine whether nutrients delivered through main ingredient

* Category 8: Meals (combination of items served as meal

replace kcalpass = 0 if eupledge_category == "8" & kcal_serving <= 340 & mealtype == "breakfast"

replace kcalpass = 1 if eupledge_category == "8" & kcal_serving > 340 & mealtype == "breakfast"

replace kcalpass = 0 if eupledge_category == "8" & kcal_serving <= 510 & mealtype == "lunch"

replace kcalpass = 1 if eupledge_category == "8" & kcal_serving > 510 & mealtype == "lunch"

replace sodiumpass = 0 if eupledge_category == "8" & sodium_mg_serving <= 660

replace sodiumpass = 1 if eupledge_category == "8" & sodium_mg_serving > 660

replace satpass = 0 if eupledge_category == "8" & satpercent <= 0.1

replace satpass = 1 if eupledge_category == "8" & satpercent > 0.1

replace sugarpass = 0 if eupledge_category == "8" & nme_sugars_serving <= 20

replace sugarpass = 1 if eupledge_category == "8" & nme_sugars_serving > 20

replace componentpass = mealcomponentpass if eupledge_category == "8"

* Category 9: Edible ices

replace kcalpass = 0 if eupledge_category == "9" & kcal_serving <= 110

replace kcalpass = 1 if eupledge_category == "9" & kcal_serving > 110

replace sodiumpass = 0 if eupledge_category == "9" & sodium_mg_serving <= 120

replace sodiumpass = 1 if eupledge_category == "9" & sodium_mg_serving > 120

replace satpass = 0 if eupledge_category == "9" & saturates_g <= 5

replace satpass = 1 if eupledge_category == "9" & saturates_g > 5

replace sugarpass = 0 if eupledge_category == "9" & total_sugars_g <= 20

replace sugarpass = 1 if eupledge_category == "9" & total_sugars_g > 20

replace componentpass = 0 if eupledge_category == "9"

* Overall pass

generate eupass = kcalpass + sodiumpass + satpass + sugarpass + componentpass

replace eupass = 1 if eupass >= 1

*Exclusions (not advertised to children under 12)

replace eupass = 1 if eupledge_category == "10"

*Exemptions (nutrient composition not assessed)

replace eupass = 0 if eupledge_category == "11"

*Drop uneccessary variables

drop kcalpass sodiumpass satpass sugarpass componentpass micropass

drop kcal_serving satpercent proteinpercent sodium_mg_serving nme_sugars_serving PUFApercent UFApercent

drop satpercentfat UFApercentenergy

*Modifications to fit format of other broadcast model .do files

generate eupnc_classification = .

replace eupnc_classification = 0 if eupass == 1

replace eupnc_classification = 1 if eupass == 0

drop eupass

**Disney model**

*Categories:

* 1 complete meal

* 2 mini meal

* 3 breakfast meal

* 4 main dish

* 5 side dish

* 6 shaped prepared nuggets and meatballs

* 7 sausages and hot dogs

* 8 cooked, cured and sliced

* 9 raw and cooked

* 10 breakfast cereal

* 11 sandwich bread

* 12 cheese

* 13 yoghurt or yoghurt-based drinks

* 14 snacks

* 15 milk (dairy/soy)

* 16 juices

* 17 water or water-based

**generate quantities per oz**

generate kcal_oz = 0

replace kcal_oz = energy_kcal/3.5274

generate serving_size_oz = 0

replace serving_size_oz = serving_size_g*0.035274

**generate calorie score**

generate disney_cal = 0

replace disney_cal = 1 if ((energy_kcal/100)*serving_size_g <= 600) & (disney_cat == 1)

replace disney_cal = 1 if ((energy_kcal/100)*serving_size_g <= 400) & (disney_cat == 2)

replace disney_cal = 1 if ((energy_kcal/100)*serving_size_g <= 600) & ((energy_kcal/100)*serving_size_g >= 400) & (disney_cat == 3)

replace disney_cal = 1 if ((energy_kcal/100)*serving_size_g <= 350) & (disney_cat == 4)

replace disney_cal = 1 if ((energy_kcal/100)*serving_size_g <= 200) & (disney_cat == 5)

replace disney_cal = 1 if (kcal_oz*3 <= 250) & (disney_cat == 6)

replace disney_cal = 1 if (kcal_oz*1 <= 80) & (disney_cat == 7)

replace disney_cal = 1 if (kcal_oz*1 <= 80) & (disney_cat == 8)

replace disney_cal = 1 if (kcal_oz*9 <= 250) & (disney_cat == 9)

replace disney_cal = 1 if (kcal_oz*1 <= 130) & (disney_cat == 10)

replace disney_cal = 1 if ((energy_kcal/100)*serving_size_g <= 150) & (disney_cat == 11)

replace disney_cal = 1 if (kcal_oz*1 <= 80) & (disney_cat == 12)

replace disney_cal = 1 if (serving_size_oz < 4) & (kcal_oz <= 30) & (disney_cat == 13)

replace disney_cal = 1 if (serving_size_oz > 4) & (kcal_oz <= 120) & (disney_cat == 13)

replace disney_cal = 1 if (serving_size_oz > 6) & (kcal_oz <= 170) & (disney_cat == 13)

replace disney_cal = 1 if (kcal_oz*1 <= 150) & (disney_cat == 14)

replace disney_cal = 1 if (kcal_oz*8 <= 150) & (disney_cat == 15)

replace disney_cal = 1 if (kcal_oz*8 <= 140) & (disney_cat == 16)

replace disney_cal = 1 if (kcal_oz*8 <= 20) & (disney_cat == 17)

**generate saturated fat score**

generate sats_per100cal = ((saturates_g/energy_kcal)*100)

generate sats_per_portion_disney = ((saturates_g/3.5274)*(serving_size_oz))

generate disney_sat = 0

replace disney_sat = 1 if (sats_per100cal <= 1.1) & (disney_cat == 1)

replace disney_sat = 1 if (sats_per100cal <= 1.1) & (disney_cat == 2)

replace disney_sat = 1 if (sats_per100cal<= 1.1) & (disney_cat == 3)

replace disney_sat = 1 if (sats_per100cal<= 1.1) & (disney_cat == 4)

replace disney_sat = 1 if (sats_per100cal <= 1.1) & (disney_cat == 5)

replace disney_sat = 1 if (sats_per100cal <= 1.1) & (disney_cat == 6)

replace disney_sat = 1 if (sats_per_portion_disney <= 1.5) & (disney_cat == 7)

replace disney_sat = 1 if (saturates_g <= 0.15) & (disney_cat == 8)

replace disney_sat = 1 if (saturates_g <= 0.15) & (disney_cat == 9)

replace disney_sat = 1 if (sats_per100cal <= 1.1) & (disney_cat == 10)

replace disney_sat = 1 if (sats_per100cal<= 1.1) & (disney_cat == 11)

replace disney_sat = 1 if disney_cat == 12

replace disney_sat = 1 if (serving_size_oz < 4) & (sats_per_portion_disney <= 1) & (disney_cat == 13)

replace disney_sat = 1 if (serving_size_oz > 4) & (sats_per_portion_disney <= 1.5) & (disney_cat == 13)

replace disney_sat = 1 if (serving_size_oz > 6) & (sats_per_portion_disney <= 2) & (disney_cat == 13)

replace disney_sat = 1 if (sats_per100cal <= 1.1) & (disney_cat == 14)

replace disney_sat = 1 if (sats_per_portion_disney <= 2) & (disney_cat == 15)

replace disney_sat = 1 if (saturates_g == 0) & (disney_cat == 16)

replace disney_sat = 1 if (saturates_g == 0) & (disney_cat == 17)

**generate sugar score**

generate disney_sugar = 0

generate added_sugars_per100cal = ((non_milk_extrinsic_sugars/energy_kcal)*100)

generate sugars_per100cal = ((total_sugars_g/energy_kcal)*100)

generate added_sugars_per_portion_disney = ((non_milk_extrinsic_sugars/3.5274)* serving_size_oz)

generate sugars_per_portion_disney = ((total_sugars_g/3.5274)* serving_size_oz)

generate added_sugars_per_1oz = ((non_milk_extrinsic_sugars/100)*28.3495)

generate sugars_per_1oz = ((total_sugars_g/100)*28.3495)

replace disney_sugar = 1 if (added_sugars_per100cal <= 2.5) & (disney_cat == 1)

replace disney_sugar = 1 if (added_sugars_per100cal <= 2.5) & (disney_cat == 2)

replace disney_sugar = 1 if disney_cat == 3

replace disney_sugar = 1 if (added_sugars_per100cal <= 2.5) & (disney_cat == 4)

replace disney_sugar = 1 if (sugars_per100cal <= 2.5) & (disney_cat == 5)

replace disney_sugar = 1 if (added_sugars_per100cal <= 2.5) & (disney_cat == 6)

replace disney_sugar = 1 if disney_cat == 7

replace disney_sugar = 1 if disney_cat == 8

replace disney_sugar = 1 if disney_cat == 9

replace disney_sugar = 1 if (sugars_per_portion_disney <= 10) & (disney_cat == 10)

replace disney_sugar = 1 if (sugars_per_portion_disney <= 5) & (disney_cat == 11)

replace disney_sugar = 1 if (sugars_per_portion_disney <= 3) & (disney_cat == 12)

replace disney_sugar = 1 if (serving_size_oz < 4) & (sugars_per_1oz <= 4) & (disney_cat == 13)

replace disney_sugar = 1 if (serving_size_oz > 4) & (sugars_per_portion_disney <= 15) & (disney_cat == 13)

replace disney_sugar = 1 if (serving_size_oz > 6) & (sugars_per_portion_disney <= 23) & (disney_cat == 13)

replace disney_sugar = 1 if (sugars_per100cal <= 6.25) & (disney_cat == 14)

replace disney_sugar = 1 if (sugars_per_1oz <= 3) & (disney_cat == 15)

replace disney_sugar = 1 if (added_sugars_per_portion_disney == 0) & (disney_cat == 16)

replace disney_sugar = 1 if (sugars_per_portion_disney <= 5) & (disney_cat == 17)

**generate sodium score**

generate disney_sodium = 0

generate disney_sodium_per_serving = (sodium_mg/3.5274)*serving_size_g

generate disney_sodium_per_1oz = ((sodium_mg/100)*28.3495)

replace disney_sodium = 1 if (disney_sodium_per_serving <= 740) & (disney_cat == 1)

replace disney_sodium = 1 if (disney_sodium_per_serving <= 600) & (disney_cat == 2)

replace disney_sodium = 1 if (disney_sodium_per_serving <= 600) & (disney_cat == 3)

replace disney_sodium = 1 if (disney_sodium_per_serving <= 600) & (disney_cat == 4)

replace disney_sodium = 1 if (disney_sodium_per_serving <= 300) & (disney_cat == 5)

replace disney_sodium = 1 if (disney_sodium_per_serving <= 480) & (disney_cat == 6)

replace disney_sodium = 1 if (disney_sodium_per_1oz <= 200) & (disney_cat == 7)

replace disney_sodium = 1 if (disney_sodium_per_serving <= 350) & (disney_cat == 8)

replace disney_sodium = 1 if (disney_sodium_per_serving <= 350) & (disney_cat == 9)

replace disney_sodium = 1 if (disney_sodium_per_serving <= 200) & (disney_cat == 10)

replace disney_sodium = 1 if (disney_sodium_per_serving <= 240) & (disney_cat == 11)

replace disney_sodium = 1 if (disney_sodium_per_serving <= 200) & (disney_cat == 12)

replace disney_sodium = 1 if (disney_cat == 13)

replace disney_sodium = 1 if (disney_sodium_per_serving <= 220) & (disney_cat == 14)

replace disney_sodium = 1 if (disney_cat == 15)

replace disney_sodium = 1 if fat_g == 0 & (disney_cat == 15)

replace disney_sodium = 0 if fat_g < 1 & (disney_cat == 15)

replace disney_sodium = 1 if (sodium_mg == 0) & (disney_cat == 16)

replace disney_sodium = 1 if (sodium_mg == 0) & (disney_cat == 17)

**generate added trans fat score**

generate disney_trans = 0

replace disney_trans = 1 if trans_fa == 0

**Add approved food count for cats 1-5**

generate approved_food_score = 1

replace approved_food_score = 0 if (disney_cat == 1) & (disney_approved_food_count <= 2)

replace approved_food_score = 0 if (disney_cat == 2) & (disney_approved_food_count <= 1)

replace approved_food_score = 0 if (disney_cat == 3) & (disney_approved_food_count <= 1)

replace approved_food_score = 0 if (disney_cat == 4) & (disney_approved_food_count <= 1)

replace approved_food_score = 0 if (disney_cat == 5) & (disney_approved_food_count <= 0)

*score*

generate disney_final = disney_trans + disney_sodium + disney_sugar + disney_sat + disney_cal + approved_food_score

replace disney_final = 0 if disney_final <= 5

replace disney_final = 1 if disney_final == 6

*labels*

generate disney_classification = disney_final

label define disney_classification 1 "approved" 0 "not_approved"

label values disney_classification disney_classification

*Drop redundant variables*

drop disney_final approved_food_score disney_trans disney_sodium

drop disney_sat disney_cal serving_size_oz kcal_oz disney_sugar

drop sats_per100cal sats_per_portion_disney added_sugars_per100cal sugars_per100cal

drop added_sugars_per_portion_disney sugars_per_portion_disney added_sugars_per_1oz

drop disney_sodium_per_serving disney_sodium_per_1oz sugars_per_1oz

** Danish model on Advertising database**

*Categories:

* 1 Dairy products

* 2 Cheese products

* 3 Meat, poultry, fish and similar

* 4 Bakery products (bread, biscuits, cakes)

* 5 Cereals

* 6 Fruit and vegetables

* 7 Sauces and dressings

* 8 Beverages

* 9 Desserts, snacks and candy

* 10 Ready made meals and convenience food

**

generate danish_fat = 1 if danish_cat !=.

generate danish_sugar = 1 if danish_cat !=.

*dairy products = danish_cat == 1

replace danish_fat = 0 if (fat_g > 2.5) & (danish_cat == 1)

replace danish_sugar = 0 if (total_sugars_g > 10) & (danish_cat == 1)

*cheese products, = danish_cat == 2

replace danish_fat = 0 if (fat_g > 20) & (danish_cat == 2)

*meat, poultry, fish and similar, danish_cat == 3

replace danish_fat = 0 if (fat_g > 20) & (danish_cat == 3)

*bakery products (bread, biscuits, cakes, danish_cat == 4

replace danish_fat = 0 if (fat_g > 10) & (danish_cat == 4)

replace danish_sugar = 0 if (total_sugars_g > 10) & (danish_cat == 4)

*cereals, danish_cat == 5

replace danish_fat = 0 if (fat_g > 10) & (danish_cat == 5)

replace danish_sugar = 0 if (total_sugars_g > 15) & (danish_cat == 5)

*fruit and vegetables, danish_cat == 6

replace danish_fat = 0 if (fat_g > 5) & (danish_cat == 6)

replace danish_sugar = 0 if (total_sugars_g > 10) & (danish_cat == 6)

*sauces and dressings, danish_cat == 7

replace danish_fat = 0 if (fat_g > 10) & (danish_cat == 7)

*beverages, danish_cat == 8

replace danish_fat = 0 if (fat_g > 0) & (danish_cat == 8)

replace danish_sugar = 0 if (total_sugars_g > 0) & (danish_cat == 8)

*desserts, snacks and candy, danish_cat == 9

replace danish_fat = 0 if (fat_g > 5) & (danish_cat == 9)

replace danish_sugar = 0 if (total_sugars_g > 5) & (danish_cat == 9)

*ready made meals and convenience food, danish_cat == 10

replace danish_fat = 0 if (fat_g > 10) & (danish_cat == 10)

replace danish_sugar = 0 if (total_sugars_g > 10) & (danish_cat == 10)

*classification

generate danish_classification = 0 if danish_cat !=.

replace danish_classification = 1 if (danish_fat == 1) & (danish_sugar == 1)

*drop redundant variables

drop danish_fat danish_sugar

***Brazilian Model**

*Categories:

* 0 Not exempted from the model

* 1 Exempt from model

*sugar

generate brazil_sugar = 0

replace brazil_sugar = 1 if (total_sugars_g <= 15) & (wxyfm_cat == "Food")

replace brazil_sugar = 1 if (total_sugars_g <= 7.5) & (wxyfm_cat == "Drink")

*saturated fat

generate brazil_satfat = 0

replace brazil_satfat = 1 if (saturates_g <= 5) &(wxyfm_cat == "Food")

replace brazil_satfat = 1 if (saturates_g <= 2.5) &(wxyfm_cat == "Drink")

*trans

generate brazil_trans = 1

*sodium

generate brazil_sodium = 0

replace brazil_sodium = 1 if (sodium_mg <= 400)

*classification

generate brazil_score = brazil_sugar + brazil_satfat + brazil_trans + brazil_sodium

generate brazil_classification = brazil_score

replace brazil_classification = 4 if (brazillian_exempt == 1)

recode brazil_classification (4=1`healthier')(0/3=0 `less_healthy')

label values brazil_classification brazil_classification

**Drop redundant variables**

drop brazillian_exempt brazil_sugar brazil_satfat brazil_trans brazil_sodium brazil_score
